# The ovine temporal bone for otologic surgery training: a systematic review

**DOI:** 10.1007/s00405-026-10290-0

**Published:** 2026-06-10

**Authors:** João H. Z. dos Santos, Juliana G. de Araújo, André L. L. Sampaio

**Affiliations:** 1 Department of Otolaryngology, Hospital de Base do Distrito Federal, Brasília, DF Brazil; 2 Universidade de Brasília, Brasília, DF Brazil; 3 Department of Otolaryngology, Hospital Universitário de Brasília, Brasília, DF Brazil

**Keywords:** Otologic Surgery, Temporal Bone, Sheep, Anatomy, Surgical Training

## Abstract

**Objective:**

Otologic surgery requires detailed anatomical knowledge and advanced technical skills, making laboratory training relevant. The limited availability of human temporal bones has encouraged the development of alternative anatomical models. This systematic review evaluates the anatomical features, surgical access routes, and applicability of ovine temporal bone models for otologic surgical training.

**Design:**

The authors conducted a systematic review across PubMed, SciELO, and Google Scholar databases. Eligible studies described anatomical findings based on microscopic or endoscopic dissection of ovine temporal bones and/or assessed their use for surgical training. Data on anatomy, surgical approaches, performed procedures, preservation methods, and participant profiles were extracted. The review followed PRISMA guidelines, and the protocol was registered in PROSPERO.

**Results:**

Twenty-six studies met the inclusion criteria. Sheep middle- and inner-ear anatomy showed close correspondence with human structures. Retroauricular, transcanal, and retrosigmoid approaches were consistently described. A wide range of procedures including tympanoplasty, ossicular chain reconstruction, stapedotomy, facial nerve decompression, and cochlear implantation were reported using microscopic and endoscopic techniques. Studies reported reductions in operative time, improved technical accuracy, and increased participant confidence. The model was consistently described as low cost, generally available, and ethically acceptable when sourced from the food industry, although some anatomical differences from the human temporal bone have been reported.

**Conclusions:**

The ovine temporal bone is a feasible and ethically acceptable model for otologic surgical training. Its anatomical realism supports a wide range of procedures, although anatomical differences should be considered when translating findings to human surgery. These findings support the incorporation of ovine temporal bone dissection into structured otologic surgical training programs.

## Introduction

Training in otologic surgery remains a major challenge in otorhinolaryngology residency programs. The anatomical complexity and variability of middle- and inner-ear structures [1, 2], combined with the need to develop hand–eye coordination and master fine motor skills, require time and extensive surgical practice [3, 4]. The introduction of endoscopic techniques appears to enhance the learning curve [3].

Cadaveric dissection facilitates anatomical understanding and the acquisition of technical skills, and has been associated with improvements in surgical performance and outcomes [5]. Although permitted in several countries, the use of human cadavers for teaching or research purposes is often constrained by limited availability [6].

In response to these limitations, alternative training modalities have been developed, including virtual simulators [7, 8] and 3D-printed bones [9, 10], which offer important educational advantages but frequently lack anatomical variability, provide limited tactile realism, and involve higher costs [11, 12].

Several animal models have been used in surgical and experimental otology, including rats [13, 14], guinea pigs [15, 16], rabbits [17, 18], cats [19], dogs [20], pigs [21, 22], goats [23, 24] and non-human primates [25]. Ideally, an animal model should approximate human anatomy and dimensions, be cost-effective, and raise minimal bioethical concerns [19, 26].

Comparative anatomical studies suggest that middle- and inner-ear structures follow evolutionary scaling patterns, with structural dimensions increasing in proportion to overall body size across species [27]. Among large-animal models, the ovine temporal bone has been considered particularly suitable for hearing research when compared with commonly used laboratory animals [28]. Furthermore, comparative analyses of cattle, ovine, and pig temporal bones for otologic surgical training suggest that the ovine model may provide anatomical advantages for surgical practice [22]

In sheep, the middle-ear and inner-ear structures present anatomical similarities to the human ear in terms of general morphology, spatial relationships of the ossicular chain, proportional dimensions and accessibility for microsurgical approaches, although proportionally smaller, allowing the use of microsurgical instruments and endoscopes [29, 30]. Surgical procedures can be performed either in vivo [31, 32] or post-mortem, with fresh specimens readily available from butcher shops at relatively low cost [22, 33]. Additionally, a comparative surgical atlas describing endoscopic ear surgery techniques based on an ovine model has been published [30].

A previous review by Fermi et al. (2022) explored the use of ex vivo ovine models across multiple areas of otolaryngology–head and neck surgery, including ear, orbital, parotid, laryngeal, and airway procedures [34]. However, the specific anatomical characteristics and surgical applicability of the ovine temporal bone for otologic training have not been systematically synthesized.

Therefore, the present systematic review focuses specifically on the anatomical and surgical applicability of the ovine temporal bone for otologic surgical training, providing a detailed analysis of temporal bone anatomy, surgical approaches, and reported training outcomes.

The primary objective of this study is to review the literature on anatomical dissection and surgical training using the temporal bones of sheep. A secondary objective is to compile data on the ear anatomy of these animals and to identify viable surgical approaches and procedures applicable to this training model.

## Materials and methods

This study is a systematic review of the literature conducted by two independent reviewers. Screening involved the evaluation of titles and abstracts, followed by a full-text assessment of eligible articles published in English or Portuguese. Any disagreements between reviewers were resolved by consensus. Literature searches were performed in the PubMed, SciELO, and Google Scholar databases, in accordance with the Preferred Reporting Items for Systematic Reviews and Meta-Analyses (PRISMA) guidelines. The review protocol was registered in the International Prospective Register of Systematic Reviews (PROSPERO; registration ID: CRD420241105879).

Search terms were organized into three thematic blocks and combined using Boolean operators (Block 1 AND Block 2 AND Block 3), as follows:

- Block 1: *Surgical anatomy, topographical anatomy, surgical approach, otologic surgery, ear surgery.*
- Block 2: *Middle ear, ossicular chain, inner ear.*
- Block 3: *Sheep, ovine, lamb.*

The search period ranged from January 1960 to June 2025.

Studies were eligible for inclusion if they described anatomy based on microsurgical dissection or endoscopic evaluation of the ovine temporal bone. Studies relying exclusively on computed tomography, magnetic resonance imaging, or histological analysis without dissection-based anatomical data were excluded.

The included studies were classified into three categories:

1. Anatomical description studies focused on the morphological characterization of the ovine temporal bone, including dimensions, anatomical relationships, and access routes, without emphasis on surgical or performance assessment;
2. Surgical exploration: studies describing surgical procedures feasible in the ovine model without objective evaluation of participant performance;
3. Surgical training: studies in which dissection was used as an educational tool aimed at teaching and developing technical skills in residents, fellows, or senior otologists.

Anatomical description studies formed the basis for comparative morphological analysis of the ovine models. Surgical exploration and training studies were evaluated with regard to technical feasibility and the educational value of the ovine temporal bone for otologic surgical training.

Data extracted from the included studies comprised general temporal bone morphology, structural measurements, and anatomical relationships, as well as details related to dissection protocols, including specimen preservation (fresh, frozen, or formalin-fixed), number of dissections performed, use of a microscope or endoscope, level of participant training (residents, fellows, or senior specialists), procedures performed, and outcome assessments. Extracted data were organized using Microsoft Excel® (Microsoft Corp., USA).

Risk of bias was assessed using criteria adapted from the Joanna Briggs Institute (JBI) and STROBE guidelines. The following domains were analyzed: selection bias (absence of clear inclusion criteria or unspecified number of specimens); measurement bias (lack of objective or reproducible outcome measures); and selective reporting bias (incomplete description of procedures or outcomes). Expectation bias was also considered when studies relied on self-assessment without independent validation. Additional methodological aspects, including clarity of study design, reproducibility of procedures, and the presence of an external or experienced otologic surgeon as evaluator, were also analyzed.

No formal certainty-of-evidence framework (e.g., GRADE) was applied, and no quantitative synthesis or meta-analysis was performed, due to the descriptive nature and methodological heterogeneity of the included studies.

## Results

**Fig. 1:**
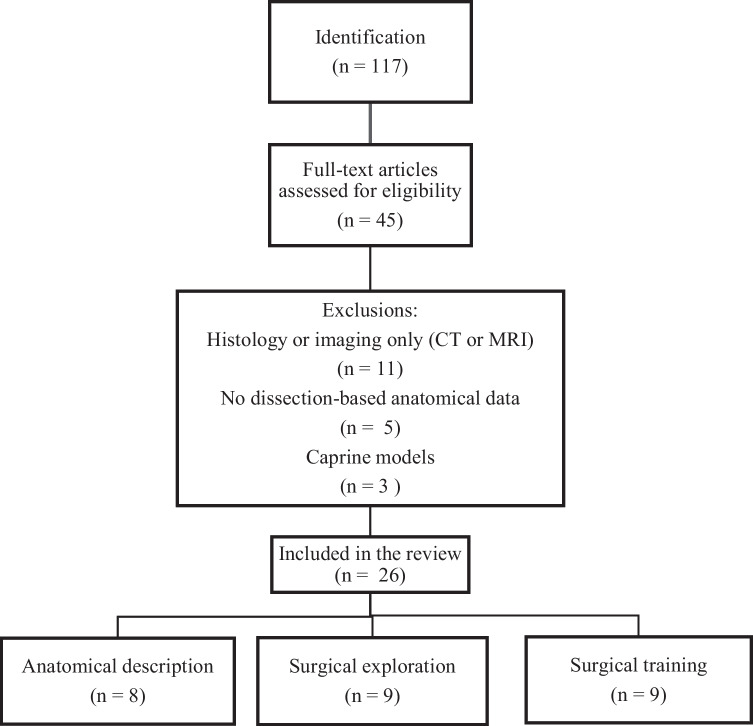
PRISMA flow diagram of study selection

A total of 117 studies were initially identified through the database searches. After removal of duplicates and title and abstract screening, 45 studies were assessed in full-text. Following full-text review, 11 studies were excluded because they presented exclusively histological or imaging-based data (computed tomography or magnetic resonance imaging), 5 were excluded due to insufficient technical detail or lack of standardized dissection description and 3 were excluded because they focused on the goat model, which was outside the scope of the present review. Consequently, 26 studies met the inclusion criteria (Table 2). Of these, 9 were classified as surgical training studies, 9 as surgical exploration studies, and 8 as anatomical description studies. The study selection process is summarized in the PRISMA flow diagram (Fig. 1).

### Anatomy

In sheep, the mastoid is spongy and trabeculated, filled with adipose and hematopoietic tissue, and poorly pneumatized [29, 35].

The external auditory canal (EAC) is tortuous, initially narrow and gradually expanding inferiorly. Its length ranges from 10.0 to 12.0 mm [35–38].

The tympanic cavity is enclosed by a smooth bony structure known as the tympanic bulla. This cavity is divided into an upper bulla and a lower bulla by the mesotympanic membrane, which extends from the incudostapedial joint to the upper portion of the promontory [35, 39].

The tympanic membrane (TM) is circular, forms an approximately 30° angle with the EAC, and lacks a clearly defined annulus [35, 37, 40, 41].

The malleus extends toward the lower bulla [37, 42, 43]. The incus presents a large convex body with two diverging processes. The long process lies close to the malleus handle [37, 42, 43]. The footplate forms an angle greater than 30° relative to the promontory [37, 42, 43].

The facial nerve is thick and frequently dehiscent within the tympanic cavity [23, 44].

The dimensional measurements of middle- and inner-ear structures reported across the included studies are summarized in Table 1.

**Table 1:** Dimensions of middle and inner ear structures in ovine and humans

Structure	Ovine	Human	Ratio	References
External auditory canal diameter (mm)	2.9–6.9	7–9	0.32–0.99	[35–38]
Tympanic membrane (mm)	8.2 × 5.3	8.5–10 × 9–10	0.60–0.85	[35, 37, 41]
Tympanic membrane area (mm^2^)	34.9–36.4	55–65	0.56–0.66	[37, 40]
Malleus (length, mm)	5.6–7.9	8.1	0.69–0.97	[37, 42, 43]
Incus (length, mm)	2.8–5.1	7.1	0.39–0.72	[37, 42, 43]
Incus (long process, mm)	2.3–3.9	3.5	0.66–1.11	[37, 42]
Stapes (height, mm)	1.6–2.1	3.4	0.47–0.62	[37, 42, 43]
Stapes footplate diameter (mm)	1.1–2.1	3.0–3.2	0.34–0.70	[42, 43]
Cochlear height (mm)	3.4–3.7	5.0–5.5	0.62–0.74	[36, 41]
Cochlear spiral length (mm)	32–34	34–36	0.91–0.97	[36, 46]
Number of turns	2.3–2.5	2.5–2.75	0.84–1.00	[36, 40]
Round window area (mm^2^)	1.8–2.5	2.7	0.67–0.93	[40, 41, 43, 44]
Oval window area (mm^2^)	2.0–2.6	3.0–3.5	0.65–0.80	[42–44]

*Note.* Ranges indicate the minimum and maximum values reported among studies. *Major diameter × minor diameter

### Surgical Approaches

The retroauricular (posterior auricular or transmastoid) approach is described as drilling the cortical bone between the temporal line and the posterior wall of the EAC, including the entire mastoid plane, to access the tympanic bulla [35, 36, 38, 39].

The transcanal approach is reported for both microscopic and endoscopic techniques and may require canalplasty to allow adequate insertion of surgical instruments [41, 44].

The retrosigmoid approach is described as a posterior fossa craniotomy that provides access to the internal auditory canal, the cerebellopontine angle, and the facial and vestibulocochlear nerves [45].

### Procedures

The reviewed studies reported the performance of ventilation tube insertion, tympanoplasty (overlay and butterfly techniques), canalplasty, ossicular chain reconstruction (OCR), stapedotomy [24, 33, 41, 46, 47], middle-ear prosthesis implantation [36], facial nerve decompression (FND) [24, 41], and cochlear implantation (CI) [32, 38, 48].

Table 2 summarizes the studies classified as surgical exploration and surgical training, detailing the surgical approaches employed, specimen characteristics, participant profiles, procedures performed, and reported outcomes.

**Table 2:** Studies using the ovine model for surgical exploration or training

Author and classification	Surgical approach	Specimens	Participants	Procedures performed	Results and conclusions	Model limitations
Lavinsky et al., 2000 [39] Exploration	Microscope	6 fresh ears	Senior otologists (N not reported)	Anatomical microsurgical exploration	Viable model with good anatomical similarity. Not suitable for mastoidectomy	Absent antrum, non-pneumatized mastoid, absence of lenticular process
Gurr et al., 2011 [35]. Exploration	Microscope	12 formalin-preserved ears	Not reported	Mastoidectomy and transcanal approach	Adequate for middle-ear access and ossicular chain manipulation	Small temporal bone, narrow EAC, non-pneumatized mastoid, absent sigmoid sinus
Schnabl et al., 2012 [36] Exploration	Microscope and endoscope	4 fresh heads	Senior otologists (N not reported)	Mastoidectomy, CI and middle-ear prosthesis (Vibrant Soundbridge)	Suitable for CI and middle-ear prosthesis	Non-pneumatized mastoid
Sudhakara Rao et al., 2019 [23] Exploration	Microscope	3 formalin-preserved ears	Not reported	Anatomical microsurgical exploration	Ovine model more suitable for initial dissection than swine and bovine models	Position of the incudostapedial joint, stapes superstructure, and oval window
Kaufmann et al., 2020 [32] Exploration	Microscope	8 live sheep	Senior otologists (N not reported)	Mastoidectomy and CI	Feasible for CI training, unsuitable for mastoidectomy	Difficulty inserting electrodes
Trinh et al., 2022 [38] Exploration	Microscope	20 fresh ears	Senior otologists (N not reported)	Mastoidectomy and CI	Excellent applicability for surgical access and CI training	Non-pneumatized mastoid, small cochlea
Waring et al., 2023 [41] Exploration	Microscope	10 fresh ears	Not reported	Extended mastoidectomy, antrostomy and CI	Viable for transmastoid approaches despite anatomical limitations	Non-pneumatized mastoid, low tegmen, narrow EAC, need to sacrifice the chorda tympani
Waring et al., 2024 [52] Exploration	Microscope	16 fresh ears	Not reported	Extended mastoidectomy, round window exposure and CI	Hampshire sheep showed greater round window visibility (83%) than Suffolk-Dorset sheep (59%)	Incomplete electrode insertion due to narrowing of the scala tympani
Korotkov et al., 2024 [47] Exploration	Microscope	5 heads formalin-preserve and silicone-injected	Senior neurosurgeon	Combined retrosigmoid and transmeatal approaches	Appropriate for retrosigmoid approach training, with good anatomical exposure	Narrow EAC, thicker and shorter vestibular and facial nerves, jugular foramen without jugular vein and absent sigmoid sinus
Gocer et al., 2007 [48] Training	Microscope	20 fresh ears	2 residents (PGY1)	Stapedotomy with Teflon prosthesis	Reduction in surgical time (from 25.2 to 16.8 min), technical feasibility, and low cost, and fewer failures	Non-pneumatized mastoid, mobile footplate
Cordero et al., 2011 [45] Training	Microscope	20 fresh ears	2 residents (PGY1)	Stapedotomy with platinum prosthesis	Average time reduced from 79 to 39.5 min and fewer complications	Small temporal bone, narrow EAC, non-pneumatized mastoid, difficult labyrinth drilling
Cordero et al., 2015 [33] Training	Microscope and endoscope (0° and 30°)	20 fresh ears	2 otolaryngologists without endoscopic experience	Endoscopic stapedectomy with platinum prosthesis	Time reduced from 38 to 31.5 min and technical improvement in stapes fracture handling	Small temporal bone, non-pneumatized mastoid, mobile footplate, difficult fenestration and crimping
Mantokoudis et al., 2016 [49] Training	Microscope	7 formalin-preserved ears	4 otologists	Mastoidectomy and cochlear implantation	Average time of 31 min. 100% success in electrode insertion. Unsuitable for mastoidectomy and posterior tympanotomy	Non-pneumatized mastoid, small cochlea, need for facial nerve manipulation and removal of the labyrinthine block
Anschuetz et al., 2017 [56] Training	Endoscope (0° and 45°) with holder	20 ears (fresh or frozen)	2 senior otologists	Tympanoplasty, incudostapedial disarticulation and OCR	Time reduced from 46 to 16 min. Subjective assessment indicated good applicability and anatomical realism	Absent annulus
Okhovat et al., 2019 [12] Training	Endoscope	1 ear	5 senior otologists and 5 residents	Endoscopic tympanoplasty; comparison between human, ovine, and synthetic bones	Ovine model superior to synthetic in realism and anatomical fidelity	Narrow EAC, large pars flaccida, and inferior position of the chorda tympani
Fernandez et al., 2021 [44] Training	Endoscope (0°)	16 frozen ears	Residents and senior otologists (N not reported)	Simulated rescue scenarios in stapedectomy (floating footplate, footplate fracture, incus dislocation, etc.). Laser use	Valid model for salvage scenarios, with good subjective evaluation	Absent annulus, small incus, absent stapedius tendon, dehiscent facial nerve, narrow EAC, difficult crimping
Beckmann et al., 2021 [46] Training	Endoscope	5 fresh ears	1 senior otologist, 1 fellow, and 1 resident	Endoscopic stapedectomy using diode laser and Teflon prosthesis	Time reduced for residents (from 42 to 21 min) and fellow (from 27 to 21 min), with fewer complications. Good anatomical realism	Summary does not detail anatomical limitations
Shrivastava et al., 2022 [3] Training	Endoscope (0°) with holder	6 fresh ears	1 fellow	Tympanoplasty, canalplasty, OCR, FND, stapedotomy and CI	Time reduced from 7 h to 3.5 h, with a positive learning curve	Absent annulus, angled footplate

*Note.* CI = cochlear implantation, Exploration = surgical exploration, Training = surgical training, FND = facial nerve decompression, N = number of participants, OCR = ossicular chain reconstruction

### Bias

A detailed summary of the risk-of-bias assessment is presented in Table 3. Among the 18 studies on surgical exploration or training, the presence of an external or experienced evaluator was reported in 14 studies (77.8%). Expectation bias was observed in 8 studies (44.4%). Only one study demonstrated a high risk of selection bias [11].

**Table 3:** Risk-of-bias assessment of included studies adapted from the Joanna Briggs Institute (JBI) and STROBE tools

Author	Selection bias	Measurement bias	Selective reporting bias	Expectancy bias	Clarity of methodology	External evaluator
Lavinsky et al., 2000 [39]	L	L	L	L	L	Yes
Gurr et al., 2011 [35]	L	H	H	N/E	L	No
Schnabl et al., 2012 [36]	L	L	M	N/E	L	Yes
Sudhakara Rao et al., 2019 [23]	L	H	H	N/E	L	No
Kaufmann et al., 2020 [32]	L	L	M	N/E	L	Yes
Trinh et al., 2022 [38]	L	L	M	N/E	L	Yes
Waring et al., 2023 [41]	L	L	L	N/E	L	Yes*
Waring et al., 2024 [52]	L	M	L	N/E	L	No
Korotkov et al., 2024 [47]	L	L	M	N/E	L	Yes
Gocer et al., 2007 [48]	L	H	H	N/E	L	No
Cordero et al., 2011 [45]	L	L	L	L	L	Yes
Cordero et al., 2015 [33]	L	L	L	N/E	L	Yes
Mantokoudis et al., 2016 [49]	L	L	L	L	L	Yes
Anschuetz et al., 2017 [56]	L	L	L	L	L	Yes
Okhovat et al., 2019 [12]	H	L	M	L	M	Yes
Fernandez et al., 2021 [44]	L	L	L	L	L	Yes*
Beckmann et al., 2021 [46]	L	L	L	L	L	Yes*
Shrivastava et al., 2022 [3]	L	L	L	L	L	Yes

*Note.* H = high risk (significant methodological flaw or missing data), L = low risk (criterion met, high methodological reliability), M = moderate risk (incomplete description or evaluator without predefined criteria), N/E = not evaluated (domain not applicable to study design). * Indicates an evaluator external to the research team

## Discussion

Overall, the available evidence indicates that the ovine temporal bone demonstrates a high degree of anatomical correspondence with the human ear. The ossicular chain demonstrates close similarity to the human ear in terms of proportions and spatial relationships, enabling realistic ossicular manipulation. Morphometric studies indicate that most ovine middle-ear structures correspond to approximately 60–80% of the dimensions observed in humans, supporting the feasibility of using standard microsurgical instruments in this model [35–37]. Likewise, the round window membrane exhibits thickness and three-dimensional architecture comparable to those of humans, supporting its use for cochlear implantation training with a high degree of anatomical fidelity [3, 40, 42].

Other animal models have also been proposed for otologic research and surgical training, including rats, guinea pigs, chinchillas, rabbits, dogs, cats, pigs, goats, and non-human primates [19, 22, 28]. Although these models have contributed substantially to experimental otology, they often present important limitations for surgical training. Compared with rodents and rabbits, the ovine temporal bone offers larger surgical spaces and anatomical landmarks more comparable to those of humans. Porcine temporal bones differ macroscopically from human anatomy, including variations in the position of the external auditory canal and key surgical landmarks, as well as thick and fatty soft tissues that may hinder procedures such as mastoidectomy [22, 52]. In caprine models, morphometric data indicate proportionally smaller and denser ossicles, a more compact tympanic cavity, a narrower and straighter external auditory canal, and a less pneumatized mastoid, which may limit surgical maneuverability and anatomical comparability with human ear [23, 24, 53].

The reviewed literature demonstrates that a broad spectrum of otologic procedures can be performed using the ovine temporal bone, including ventilation tube insertion, tympanoplasty, OCR, stapedotomy, FND, and CI. Some studies also explored novel technologies, such as laser-assisted stapedotomy [46, 49] and middle-ear anchored prostheses [36], which remain unavailable in some residency programs. Both the retroauricular (transmastoid) and transcanal approaches are technically feasible in the ovine model, allowing the use of either a microscope or an endoscope [38, 39, 45].

Endoscopic applications were widely explored and demonstrated high feasibility for surgical training [33, 50]. The use of a holder enabling two-handed dissection allowed surgeons to practice advanced endoscopic techniques [49, 51].

Training-based studies reported well-defined learning curves, with progressive reductions in operative time and improvements in procedural performance [3, 49, 51]. Participants also reported substantial gains in confidence and technical proficiency, including during simulations of rescue situations or intraoperative complications [33, 49].

Quantitative data from six studies corroborated these findings, with reported reductions in operative time ranging from 17 to 65%. The greatest benefits were observed during the early stages of otologic training and among experienced surgeons adapting to endoscopic techniques [3, 33, 44, 46, 47, 51]. Nevertheless, it is important to recognize that a portion of the observed reduction in operative time may be attributable to increasing familiarity with ovine temporal bone anatomy and procedural repetition, rather than reflecting a full and direct transfer of motor skills to human otologic surgery.

Despite its favorable attributes, the ovine model presents relevant anatomical limitations that necessitate technical adaptations but do not compromise its overall utility for anatomical study and surgical training [22, 35]. The hypopneumatized mastoid limits classical mastoidectomy and posterior tympanotomy training [29, 35].

The reduced diameter and tortuous configuration of the EAC often require extensive canalplasty to allow adequate insertion of instruments and endoscopes [51]. Although this represents a technical challenge, it also provides an opportunity to develop spatial awareness and fine motor control.

The tympanic membrane lacks a fibrous annulus, increasing the risk of lacerations to the membrane and tympanomeatal flap [23, 33]. The stapes and footplate, which are smaller and oriented at less favorable angles, make fenestration technically demanding. The fragility of the stapes superstructure and the limited working space between the footplate and the incus increase the risk of fractures or unsuccessful stapedotomy [33, 46, 49]. The facial nerve is frequently dehiscent, requiring heightened caution during dissection, particularly in endoscopic approaches [1, 5].

Logistically, the ovine model is accessible, with specimens obtained from slaughterhouses or specialized suppliers at low cost. Temporal bones can be used fresh or frozen while preserving key anatomical characteristics necessary for training [39, 44].

Ethically, the use of ovine heads obtained from the food industry is generally considered acceptable under prevailing regulatory frameworks and typically does not require formal ethics approval. The use of animal-derived specimens may also raise cultural or religious considerations in some contexts, particularly in institutions with specific ethical or religious frameworks.

This review also highlights limitations inherent to the existing literature. Only Beckmann et al. (2021) and Fernandez et al. (2021) combined structured self-assessment tools with independent expert evaluation. Okhovat et al. (2019) used a validated Likert scale without external raters, while Shrivastava et al. (2022) employed descriptive self-assessment supervised by a senior surgeon. Among surgical exploration studies, only Waring et al. (2023) included a truly independent evaluator.

Anatomical variations may exist among different sheep breeds. Waring et al. demonstrated that Hampshire sheep present improved round window visibility compared with Suffolk-Dorset specimens, likely due to differences in the trajectory of the facial nerve relative to the round window [54]. These findings suggest that breed-specific anatomical variability may influence surgical accessibility and should be considered. However, there are insufficient data in the literature to support the choice of one species over another.

Considerable methodological heterogeneity was observed regarding surgical approaches, number of dissections, outcome measures, and participant profiles, precluding meta-analysis and potentially introducing selection, reporting, and expectation biases. In addition, most studies were conducted in specialized centers with experienced participants, which may limit generalizability to other training settings. A potential limitation of the review process is the inclusion of Google Scholar, which may increase screening workload; however, selection was performed independently by two reviewers.

## Conclusion

This systematic review indicates that the ovine model is a viable alternative for training in otologic surgery. The anatomy of the middle and inner ear offers a good correspondence with human structures, allowing the performance of procedures such as tympanoplasty, canalplasty, OCR, stapedotomy, and CI.

Despite its limitations—such as a narrow EAC, the absence of a tympanic annulus, and a hypopneumatized mastoid—the model permits training in advanced techniques, including simulations of intraoperative rescue situations and the use of laser-assisted techniques and hearing devices. Ovine heads are generally accessible, relatively inexpensive, and, when sourced from the food industry, typically exempt from additional ethical requirements.

Although the ovine temporal bone does not fully replicate the complexity of human temporal bone anatomy, it represents one of the most practical, accessible and reproducible animal models currently available for structured otologic surgical training.
